# Adaptation of human skin color in various populations

**DOI:** 10.1186/s41065-017-0036-2

**Published:** 2017-06-15

**Authors:** Lian Deng, Shuhua Xu

**Affiliations:** 1 Chinese Academy of Sciences (CAS) Key Laboratory of Computational Biology, Max Planck Independent Research Group on Population Genomics, CAS-MPG Partner Institute for Computational Biology (PICB), Shanghai Institutes for Biological Sciences, CAS, Shanghai, 200031 China; 2 University of Chinese Academy of Sciences, Beijing, 100049 China; 3 School of Life Science and Technology, ShanghaiTech University, Shanghai, 201210 China; 4 Collaborative Innovation Center of Genetics and Development, Shanghai, 200438 China

**Keywords:** Skin color, Natural selection, Genetic adaptation, Modern humans, Archaic hominin

## Abstract

**Background:**

Skin color is a well-recognized adaptive trait and has been studied extensively in humans. Understanding the genetic basis of adaptation of skin color in various populations has many implications in human evolution and medicine.

**Discussion:**

Impressive progress has been made recently to identify genes associated with skin color variation in a wide range of geographical and temporal populations. In this review, we discuss what is currently known about the genetics of skin color variation. We enumerated several cases of skin color adaptation in global modern humans and archaic hominins, and illustrated why, when, and how skin color adaptation occurred in different populations. Finally, we provided a summary of the candidate loci associated with pigmentation, which could be a valuable reference for further evolutionary and medical studies.

**Conclusion:**

Previous studies generally indicated a complex genetic mechanism underlying the skin color variation, expanding our understanding of the role of population demographic history and natural selection in shaping genetic and phenotypic diversity in humans. Future work is needed to dissect the genetic architecture of skin color adaptation in numerous ethnic minority groups around the world, which remains relatively obscure compared with that of major continental groups, and to unravel the exact genetic basis of skin color adaptation.

## Background

Since modern humans ventured out of Africa ~100,000 years ago, they spread across continents into a variety of habitats, from tropical zones to the arctic, and from lowlands to highlands. During migration, selective pressures in local environments (e.g., the cold climate, hypoxia, and endemic pathogens), together with random drift, have resulted in population-specific genetic variants, which further influenced variable phenotypes, such as lactose tolerance, height, immune system, and metabolic efficiency.

Skin color variation is one of the most striking examples of human phenotypic diversity. It is dominated by melanin, a pigmentation located in the base of the epidermis and produced by melanocytes. Melanin has two forms, pheomelanin (yellow-reddish) and eumelanin (black-brown). The former is mainly accumulated in the light-complexioned people, while the latter is mostly produced in the dark-complexioned people [1–5]. In addition, the number and size of melanin particles differ among individuals, and is even more important than the proportions of the two forms of melanin in the determination of human skin color [5]. Other skin-related factors, e.g., keratin, also contribute to skin color variation [6, 7].

**Fig. 1:**
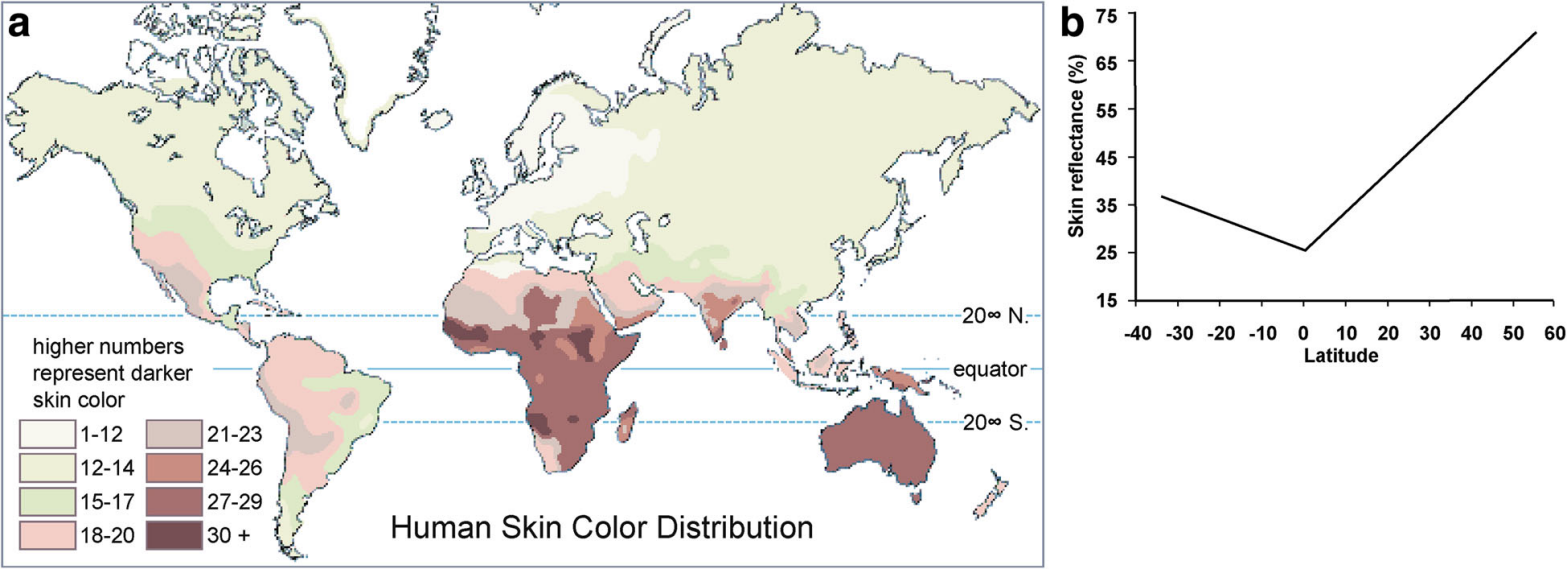
Correlation between skin color and latitude (from Barsh (2003) [5]). (**a**) A map of human skin color distribution. (**b**) A plot of skin reflectance against latitude

In global populations, skin color is highly correlated with latitude, and fundamentally, the distribution of ultraviolet (UV) radiation (Fig. 1). Populations closer to the equator tend to have dark skin for protection against UV, since overexposure to UV may decrease folic acid levels [8, 9] and cause skin cancer [10–13]. The lighter skin in populations at higher latitudes is underlying selection to maintain vitamin D photosynthesis, which is a UV-dependent process [14, 15].

Although UV has been assumed to be a driving force for the evolution of human skin colors, understanding the exact genetic mechanism of selection would be crucial to reconstruct human evolutionary history and elucidate the microevolution of adaptive traits. Describing a full picture of regional skin color adaptation in humans would be challenging because it includes not only the genes identified to be under selection, but also the extent to which these genes could explain phenotypic variation, the interactions and joint effects of genes, and the way they react to the external environments. In this article, we reviewed several cases of skin color adaptation in various populations of modern humans and archaic hominins. These cases show the similarities and differences of mechanisms of skin color adaptation across populations, and provide some insights into human evolutionary history.

### Skin color adaptation in modern Eurasians

**Fig. 2:**
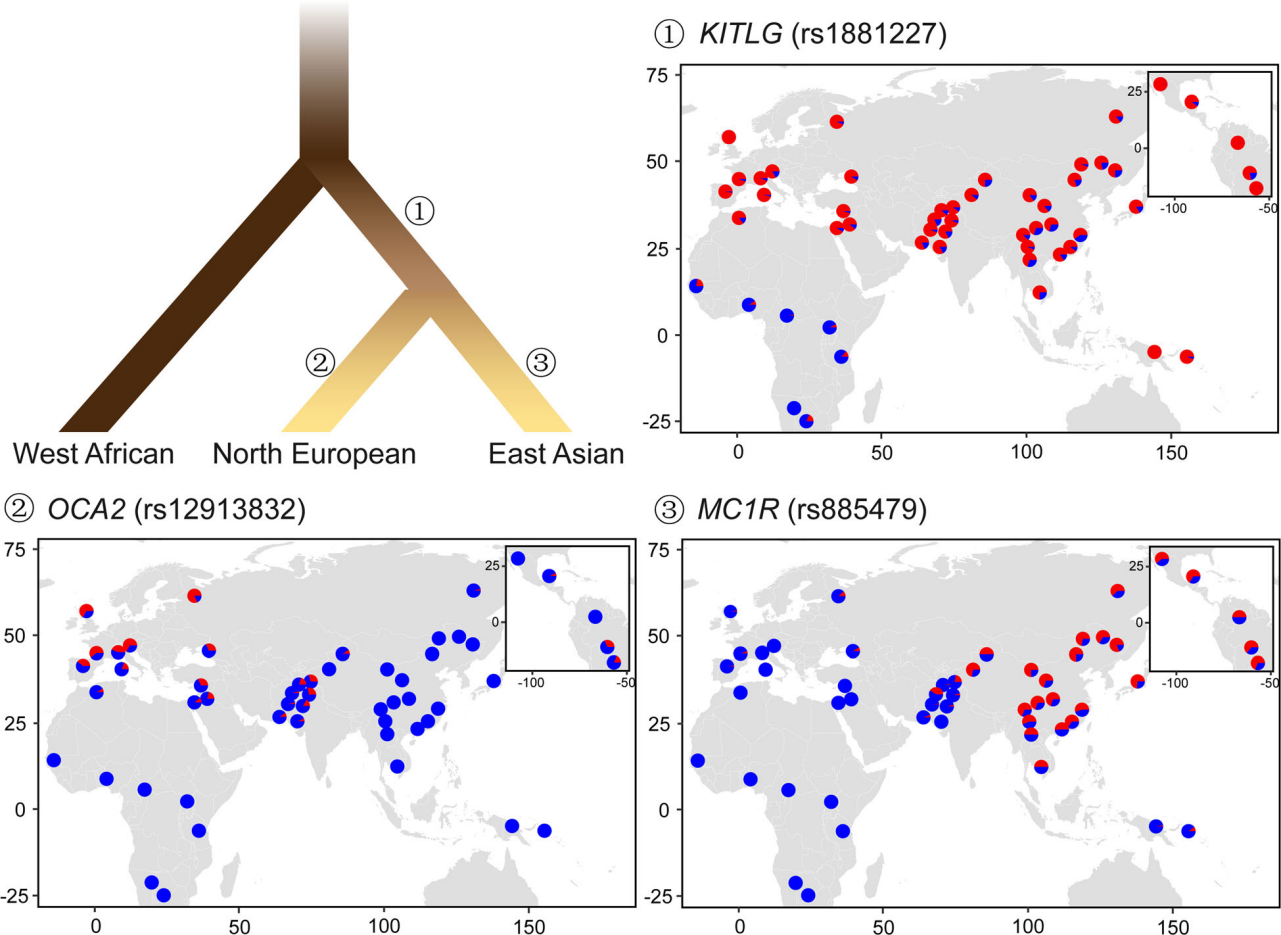
Evolutionary model of human pigmentation in three continental populations. The rooted tree shows the genetic phylogeny of human populations from Africa, North Europe and East Asia, with the colors of the branches roughly indicating the generalized skin pigmetation level of these populations (adapted from McEvoy et al. (2006) [39]). Genetic loci reported to be under positive selection in the common ancestor of modern Eurasians are represented by rs1881227 in *KITLG*, and those independently evolved in Europeans and East Asians, indicating possible convergent evolution, are represented by rs12913832 in *OCA2* and rs885479 in *MC1R*, respectively. The maps of allele frequency were drawn using *R* (version 3.2.1, https://www.r-project.org), based on these loci in 53 global populations provided by the Human Genome Diversity Panel CEPH (HGDP, http://www.hagsc.org/hgdp/index.html). Blue and red colors denote the ancestral and derived alleles, respectively

In Europeans, *SLC24A5* and *SLC45A2* [16–19] are two golden genes related to the evolution of the light skin color. *SLC24A5* encodes the NCKX5 protein, which is a member of the transmembrane protein family and regulates the calcium concentration in the melanosome [16]. This gene has been confirmed to affect pigmentation in zebrafish and mice [16, 20]. Especially, the derived allele of rs1426654 in *SLC24A5* was found to be nearly fixed in Europeans, but almost missing in populations without any European ancestry (Fig. 2) [21]. A 78-kb haplotype around *SLC24A5*, which is in linkage disequilibrium with rs1426654, was also identified to accumulate in Europeans [22]. A similar pattern can be observed at rs16891982 in *SLC45A2* [23], which has been reported to be associated with pigmentation in several species, e.g., mice, fish, birds, and horses [24–26]. Other variants in this gene, including rs26722, rs2287949, and rs40132, were also shown to be coloration-associated in Europeans [23, 27, 28]. Another important pigmentation-related gene identified in European is *MC1R* [29–31]. This gene is expressed in melanocytes and plays a key role in controlling the switch from pheomelanin to eumelanin [31]. The pigmentary phenotypes associated with *MC1R* has been studied in a wide range of animals [32–34]. Many variants have been identified in *MC1R*, such as rs1805007, rs1805008, and rs3212357 [35, 36], despite its small size (951 bp). Other important European-specific loci include rs1393350 in *TYR*, rs2733831 in *TYRP1,* and rs1900758 in *OCA2* [17, 28, 37–39]. The derived allele frequencies at these loci are high in Europeans but low in Africans and East Asians, which could be a clear signal of positive selection in Europeans, as indicated by statistical analysis [40].

Genes involved in the skin color adaptation in East Asians are not that well studied compared to the long list of adaptive genes identified in Europeans. Notable examples include *OCA2* and *MC1R*. Each harbors several non-synonymous mutations, e.g. rs1800414 and rs74653330 in *OCA2,* and rs885479 in *MC1R* [40–43], which exhibit high derived allele frequencies in East Asians, but low derived allele frequencies in Europeans and Africans (Fig. 2). The OCA2 protein is thought to be a mature melanosomal membrane protein [44], with a potential role in protein transportation into melanosomes [45]. The East Asian-specific variant of rs1800414 was first reported in an exome sequencing study aiming to figure out albinism-related variants [46]. The derived allele at rs1800414 was thought to contribute to the skin lightening in an association study of Han Chinese, which measured the skin color of individuals using the melanin index [47]. Another non-synonymous variant in *OCA2*, rs74653330, has also been confirmed to be pigmentation-related in an association study of Japanese [48]. Additional examples of East Asian-specific pigmentation-associated alleles include rs10809814 in *TYRP1* and rs1407995 in *DCT* [40, 49], both of which show differentiation between Asians and non-Asians [47], and strong signals of positive selections in Asians [43, 49].

Despite distinct genes and variants under respective local adaptations in Europeans and East Asians, some genes have derived alleles reaching high frequencies in both continental groups. For instance, *KITLG* exhibits a selective sweep in non-Africans [50–52]. This gene is widely expressed in multiple tissues, including the skin, and functions in organ morphogenesis and cell proliferation. The Kit-ligand encoded by *KITLG* is known as the steel factor and plays a crucial role in the normal development and maintenance of the melanocyte lineage in adult skin [53]; this has been proved in human, fish, and mice [54–56]. The effects of this gene on pigmentation have also been confirmed in a series of association studies [57–60]. One of the key variants is rs642742, which is located at 326 kb upstream to the transcription start site of *KITLG*. At this variant, the ancestral allele frequency is over 90% in Africans, comparable to the derived allele frequency in Europeans and East Asians (Fig. 2). Similar patterns were observed in other genes, e.g., *ASIP* and *BNC2* [39].

Two models of the evolutionary architecture of human pigmentation were proposed on the basis of the above results and other related studies (Fig. 2). One is a convergent evolution model [17, 40, 43, 49], suggesting that depigmentation has, to some degree, evolved independently in Europeans and East Asians, as different genes and variants have been suggested to explain the light skin and positive selection in these two continental groups. A recent study estimated the time of selective sweeps for the European-specific pigmentation variants to be around 11,000–19,000 years ago, after the divergence of Europeans and Asians [61]. An alternative model fits for the shared selective sweeps of Europeans and East Asians, which could possibly occur in proto-Eurasians. The onset of the sweep was estimated to be approximately 30,000 years ago, right after the “Out-of-Africa” migration, but earlier than the European-specific evolution on pigmentation [61]. The coexistence of these two models suggests a complex evolutionary history of skin color in modern humans.

Another clue of the complex genetic basis of skin color evolution is the allelic heterogeneity observed in a single gene, like *OCA2* and *MC1R*. In each of these genes, some alleles are specific to Europeans, whereas others are specific to Asians, although they all have been proved to be depigmentation-related. In addition, *OCA2* provides evidence of independent sweeps as well as convergent evolution in Europeans and Asians. Since results were obtained from studies using different samples, data, and methods, there could be some confounding factors leading to these different observations. However, more importantly, skin color is a complex trait that could not be simply explained by a single gene or variant; rather, it is likely to involve a huge network of genes and phenotypes. For instance, *ASIP*, an adaptive pigmentation gene in populations with European ancestry [62, 63], encodes the agouti signaling protein, which blocks *MC1R* in the eumelanin synthesis in response to the UV-induced DNA damage [40]. In the melanin production, *TYR* acts as the catalyzer of the key initial step, and its stability is maintained by *TYRP1* and *DCT*.

In addition, scans for selection on skin pigmentation indicate two different selection behaviors acting on de novo mutations and standing variations, respectively. Some variants, represented by rs1805007 and rs1805008 in *MC1R* (in Europeans) and rs1800414 in *OCA2* (in Asians), only show derived alleles in populations under positive selection at these loci, from which we could conjecture that they are new mutations that appeared after modern humans settled in Europe or Asia. In contrast, some variants, such as rs3212357 in *MC1R* (under positive selection in Europeans), present low frequencies in Africans. Regardless of possible mutation events and genetic drift in African populations, it is more likely that the derived allele at this locus has presented for some time before they became favored. Similar cases have been found in the high-altitude adaptation of Tibetans and the immunity adaptation in some modern human populations, and even in the evolution of pigmentation phenotypes in non-human species [56, 64].

### Skin color adaptation in the admixed populations

Admixed populations, the hybrid offspring of two previously isolated populations, may provide important insights in understanding the genetics of geographical variation for two reasons. First, the loci underlying phenotypic differences in ancestral populations are also overlapping the highly informative markers of ancestry, which makes the admixed populations particularly useful for tracing population history. Second, the admixed populations usually have a wide range of variations regarding some specific phenotypes, which may increase the power of locating genes associated with complex traits/diseases after controlling potential population stratification.

Despite these advantages, admixed populations have rarely been considered in studies of human pigmentation variation. Current studies investigating pigmentation genes in admixed populations mainly involved those with African and European ancestry, such as African Americans, European Africans, and Latin Americans, since their ancestral populations are substantially differentiated in skin color. The ancestral genetic makeups differ among these three populations. African-Americans obtained the largest genetic contribution (~80%) from the African ancestry [65], Latin American mestizos have the least proportion of African ancestry (~10%) [66, 67], while in European Africans, the genetic components inherited from Europeans (~42%) and Africans (~58%) are comparable [68]. Uniquely in the Latin Americans, a considerable proportion of Native American ancestry (~45%) exists [66, 67]. Moreover, on the individual level, the proportion of each ancestry exhibits a large variance in each admixed population. For instance, the fraction of European ancestry varies from 2% to 98% among African American individuals [65]. The large variance of skin color in admixed individuals could result from their highly diverse genetic makeup, as a substantial correlation has been observed between ancestry proportion and skin color [68–70].

**Fig. 3:**
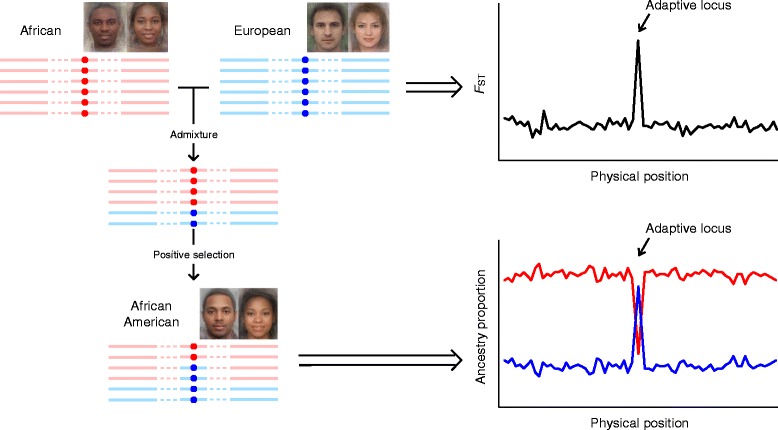
A framework of admixture mapping to detect positive selection. The average faces of African, European, and African America were downloaded from http://www.mediadump.com/hosted-id167-average-faces-from-around-the-world.html#.WLkMU-kfU1A

Multiple well-known candidate genes for pigmentation in Europeans have also been identified by admixture mapping (Fig. 3) or association studies in admixed populations. For instance, *TYR*, carrying a non-synonymous substitution rs1042602 (S192Y), was identified in African Americans [69] and European Africans from Cape Verde [68]. Variants in *ASIP,* such as rs6058017, which has been found to occur at different frequencies in global populations [63], were also reported to be associated with dark hair and brown eyes in European Americans [71], African Americans [62] and Brazilians [72]. Furthermore, *KITLG* showed strong signals of selective sweep in African Americans [51], with a significant preference to homozygotes of the African-specific allele (ancestral allele) at rs642742 in individuals with high melanin index (dark skin) [69]. Similar cases include rs1426654 in *SLC24A5* in European Africans [68] and Latin Americans [72], and rs35395 in *SLC45A2* in European Africans [68].

However, some studies reported discrepant results. The correlation between Native American ancestry and skin pigmentation reported in a Hispanic population [73] was not observed in a group of Puerto Rican women [70]. One of the key single nucleotide polymorphism loci (SNPs) in *OCA2*, rs1800404, showed a significant effect on skin pigmentation when analyzing African Americans and a combined population of African American and African-Caribbean, but was absent in an independent analysis of the African-Caribbean samples [69]. It is possible that different genetic mechanisms of skin color variation exist in various populations, but cautions should be taken regarding detailed information in the data, such as sample size and the ancestral populations selected for analyzing the admixed populations, which could lead to biased results [67, 69].

The identification of genetic determinants of natural variation of skin pigmentation was also conducted in other admixed populations. One successful example is a genome-wide association study of a population of South Asian descent [74], in which polymorphisms in *SLC24A5*, *TYR* and *SLC45A2* showed significant associations with the melanin content in skin. The light skin alleles in South Asian could possibly be inherited from their European ancestors [75], who initially arrived at this region around 3500–4000 years ago along with Indo-European language expansion [76], followed by recent colonization in the last few centuries. In addition, Central Asia and Southeast Asia are home to various admixed populations, which are likewise of great potential in the study of skin color adaptation. Admixed population analyses may greatly enrich our understanding of skin color variation in modern human populations.

### Skin color adaptation in the aboriginal populations

The aboriginal populations in different areas around the world have many implications for human evolutionary history. They have been regarded as the early settlers in respective areas. Despite having been assimilated by their surrounding agriculturalists to some extent, some aboriginal people have preserved their traditional livelihoods as hunter-gatherers, as well as their original physical traits – dark skin, short stature, and curly hair.

**Fig. 4:**
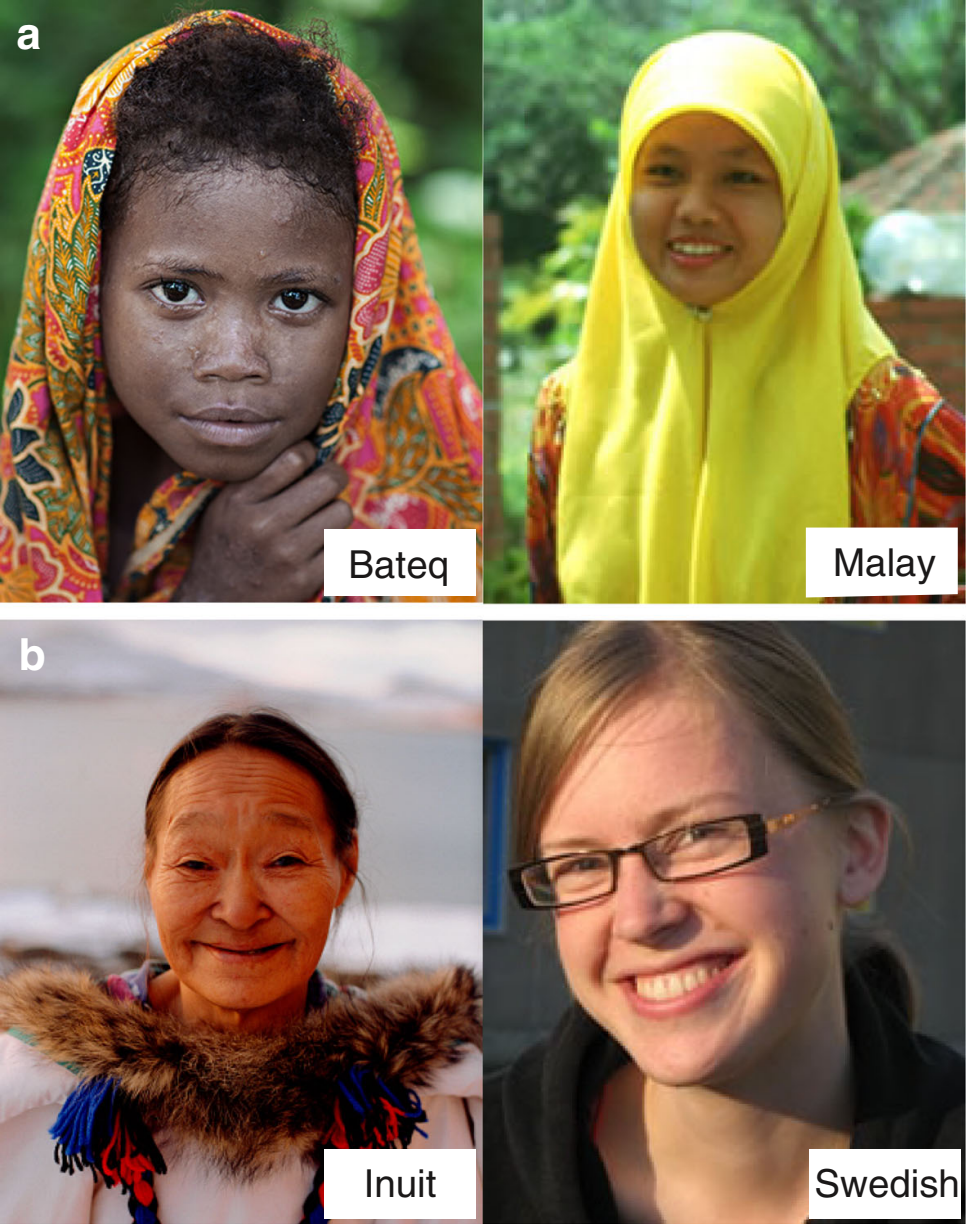
Skin color of aboriginal people in the Equatorial zone and the Arctic. (**a**) Skin color comparison between Bateq (a subgroup of Negrito) and Malay from Peninsular Malaysia. (**b**) Skin color comparison between Inuit and Swedish from similar latitudes. Portraits of Malay and Swedish individuals are provided by the Joshua Project (http://joshuaproject.net), the Bateq portrait is from http://www.businessinsider.my/, and the Inuit portrait is from http://www.arcticphoto.co.uk/

The hunter-gatherer populations with dark skin, short stature and curly hair have attracted much attention (Fig. 4a). The genetic mechanism underlying the shared phenotypes among these geographically distant populations (collectively called Negritos or Pygmies), from Central Africa, the Andaman Islands, Southeast Asia and Oceania, are still controversial; for example, whether they were the common descent from a pre-Neolithic substrate of humanity or a consequence of convergent evolution [77, 78]. To date, most genetic studies on this issue have focused on height [78–81]. One study provided clues for convergent evolution from the view of skin pigmentation adaptation by analyzing *MC1R* diversity in the Melanesians [82]. This study showed that the ancestral haplotypes of *MC1R* are not highly conserved between Northern Island Melanesians and Africans, although both populations live in the high UV region, which is in contrast to previous findings based on very limited samples [30, 83]. Besides, a non-synonymous polymorphism, rs2228479, shows enriched derived alleles specifically in East Asians, but is not significantly associated with skin or hair pigmentation in Melanesians. Actually, the Melanesian population exhibits striking skin pigmentation variation [84], and consistently, some variants have been identified to be region-specific, which could partly explain this phenotypic variation. A notable example is a non-synonymous variant, rs387907171, in *TYRP1* [85]. It is restricted to the Solomons and parts of the Bismarck Archipelago, and might contribute to the ‘blond hair’ in this region [85, 86]. These results emphasize the complex genetic architecture of pigmentation phenotypes, and also highlight the role that population history (e.g., the complex population history of the Southwest Pacific [87–89]) can play a role in influencing phenotypic diversity. Skin pigmentation studies on other modern aboriginal populations (besides Melanesians) are scarce, except for one investigating the Senoi population (an indigenous population) from the Malay Peninsula, which is an admixture of the Negrito (dark-skinned) and the southern Mongoloid from Indo-China (yellow-brown-skinned), and has a wide skin color spectrum [90]. The authors of this study found that despite the low derived allele frequency, the A111T mutation (rs1426654) in *SLC24A5* is significantly associated with the light skin in Senoi, which was suspected to result from the admixture of the Mongoloid and South Asians.

Another interesting issue concerning human skin color adaptation comes from the arctic people. The Inuit people, in far North Eastern Asia and the American Subarctic, have yellowish-brown skin despite the far northern latitude at which they live, unlike other populations living at the same latitude, such as the Swedes and Finnish (Fig. 4b). This makes the Inuit population an exception of the latitude-correlated distribution of skin color. One possible reason is that the dark skin could protect the Inuits from the severe UV exposure because of the long daylight hours in winter and high levels of UV reflection from the snow. While the dark skin is a disadvantage for vitamin D production, plenty of vitamins including vitamin D could be compensated from their diets [91, 92]. Another cause could be the founder effect of the ancient East Asian ancestry of the Inuits, who have inhabited the arctic region since nearly 5000 years ago, and had higher melanin production than the European ancestry. However, very few genetic studies have been conducted to determine the genetic basis of dark skin in arctic populations.

### Skin color adaptation in the ancient hominins

The dark skin in modern humans was established around 1.2 million years ago, driven by the loss of body hair after divergence from apes, presumably to protect against UV-induced damages [13, 93–96]. Then, when did modern Eurasians start to depigment? The studies on skin color adaptation summarized above are based on modern population genetic data, which may suffer from limited temporal resolution caused by the population demographic history, and insensitivity to selection acting on standing variations [97]. The advent of ancient DNA analyses makes it possible to directly observe the evolution processes, and thus would facilitate our understanding of this key question.

A study on the genomes of Anatolian Neolithic farmers in West Eurasia (6500–300 BC), who are probably the source population of the first European farmers, suggests that the light skin color has been evolved since at least 6500–4000 years ago [98]. Several popular genes identified in modern Eurasians, e.g., *SLC45A2*, *GRM5* and *HERC2*/*OCA2* showed strong signal of selection in these ancient samples. This conclusion is supported by another study based on the Eneolithic (6500–5000 BP) and Bronze Age (5000–4000 BP) samples, representing the early European farmers or late hunter-gatherers in central Europe [99]. One possible motivation of the skin depigmentation in prehistoric Eurasia is agriculturalization, which led to a switch from vitamin D-rich hunter-gatherer diet to a vitamin D-poor agriculturalist diet, together with the increased danger of folic acid deficiency at higher latitudes [14, 100]. Moreover, the selective pressures have kept operating for a long time after they initiated the adaptation of skin color, as some ancestral pigmentations alleles were identified in a Mesolithic European (7000 BP), and some adaptive alleles under selection in the ancient Eurasians are still evolving in modern humans [98, 99, 101].

Recent studies on archaic hominins (e.g., Neanderthals, an extinct hominid group living in Eurasia ~400,000–28,000 years ago [102]) further improved our understanding of skin color evolution in modern humans. Neanderthals met modern humans in the Middle East ~60,000–50,000 years ago, and contributed to about 1–4% of modern human genomes [103–105]. Some pigmentation-associated genes are identified in the introgressed haplotypes from Neanderthals in modern Eurasians, such as *POU2F3*, *BNC2* and *MC1R* [106, 107]. Specifically, the introgressive alleles were reported to result in light skin color, suggesting an ‘adaptive introgression’ strategy of human skin color adaptation. Other introgressive genes related to skin phenotypes include *HYAL* genes, which are associated with cellular responses to UV and are under strong positive selection in East Asians [108], and those involved in keratin filaments formation [109]. Although these genes are not direct determinants of skin pigmentation, they, like those pigmentation-related genes, possibly helped modern humans adapt to non-African environments.

When drawing conclusions of adaptive introgression, we are actually claiming that Neanderthals could be light-complexioned. This inference is just based on some pigmentation-associated genes or alleles identified in existing modern human populations, since visible phenotypes of Neanderthals and other extinct species are not available. However, when using some other priory genes as potential clues, different results can be obtained. For instance, the derived state of *MC1R*, which is responsible for pale skin, presents in Neanderthal individuals from Italy and Spain but is missing in Croatian Neanderthals and Denisova [110], suggesting skin color variation in the archaic hominins. In addition, the light skin in Neanderthals and modern Eurasians could also result from convergent evolution, rather than adaptive introgression [111].

The hypothesis of adaptive introgression seems to predate when modern human became pale – long before the late Mesolithic age, as Neanderthals went extinct around 28,000 years ago. However, we should reconsider whether the genes affecting skin color in archaic hominins indeed determined skin color in modern humans. Even if this is the case, it is also possible that modern human retained these introgressive variants until they showed some phenotypic effects under some specific strong selective pressures. Thus, more data resources and analyses are necessary to address this issue in the future.

### Selection coefficient and effect size

As one of the most obvious changes in the environment after modern human migrated out of Africa to higher latitudes, UV has exerted considerable selective pressures on human skin pigmentation, which can be reflected by selection coefficients of the pigmentation-related genes. The estimation of selection coefficients largely depends on the genes considered and the methodologies. Beleza et al. estimated the coefficient of selection at several loci representing *SLC24A5*, *SLC45A2*, *TYRP1,* and *KITLG* [61]. For example, the estimates are 0.05/0.04 for *SLC45A2* and 0.16/0.08 for *SLC24A5* under a dominant/an additive model of inheritance in Europeans. Meanwhile, López et al. reported the selection coefficient of a variant in *SLC45A2* to be 0.01–0.02 in a South European populations [112]. These estimations are comparable to the selection coefficients inferred directly from serially sampled data at *HERC2*, *SLC45A2*, and *TYR*, ranging from 0.02–0.1 [99]. The selection coefficients estimated for pigmentation genes are best understood in the context of estimates for other recently selected loci. The selection advantages are inferred to be 0.01–0.08 for *LCT*, a gene strongly associated with lactase persistence in populations with European ancestry [113, 114], 0.019–0.048 for *G6PD*, a gene conferring malaria resistance in African populations [115], 0.03–0.19 for *EDAR* associated with the increased scalp hair thickness and changed tooth morphology in the Han Chinese [116], and 0.0004–0.0023 for *EGLN1* and *EPAS1* gene regions contributing to the high-altitude adaptation in Tibetans [117]. The selection coefficients for pigmentation genes are among the most strongly selected genes in the human genome, indicating a severe selective pressure caused by UV or some other environmental changes in non-African regions.

Although a large number of genes have been identified to contribute to skin color variation, how much could they explain the skin color variation in modern humans? Is there a gene or variant that has a dominant effect on the skin color? Some genes could possibly play a major role in determining skin color in specific populations. For instance, the light skin variant at rs1426654 in *SLC24A5* could explain 22–32% of the variance of the melanin index in South Asian [75] and 25–38% in African-American and African-Caribbean populations [118]. Additionally, the derived allele at rs642742 in *KITLG* may account for lightening of a person’s skin by 6 to 7 melanin units, nearly 1/5 of the overall skin reflectance difference between West Africans and Europeans (30 melanin units) [56]. However, there are relatively more genes and variants with smaller effects. One of the key variants in *OCA2*, rs1800414, could explain around 4% of the pigmentation variation in East Asian populations [47]. In South Asians, rs16891982 in *SLC45A2* and rs1042602 in *TYR* account for 3.6% and 2.5% skin color variation, respectively, much less than the effect size of rs1426654 in *SLC24A5* [74]. The inheritance mode of skin pigmentation follows an additive model, or at least an incomplete additive model [16, 17, 47, 56, 75].

## Conclusions

Overall, human skin color is a highly variable and complex trait as a consequence of strong selection pressure and is controlled by multiple genetic loci (summarized in Table 1). Skin color adaptation is a complex process because different populations have shared and independent genetic mechanisms involving a large number of genes with different effect advantages on the phenotype. Skin color adaptation is also a long evolutionary process influenced by various historical, even pre-historical, population genetic events. Current studies provide comprehensive insights into the natural selection process and mechanisms of human skin color variation. A richer resource of high-coverage whole-genome sequences and phenotype data may provide opportunities to further speculate an accurate model of genetic architecture and gene-environment effects, and advance our understanding of skin pigmentation in certain minor ethnic groups, such as hunter-gatherers and highlanders. We believe that these studies may greatly enrich our knowledge of human evolution history and elucidate the genetic basis of complex traits in humans.

**Table 1:** A summary of candidate genes of human adaptation on pigmentation

Gene	SNP	Population	Skin color	Hair color	Eye color	References
*APBA2*	rs4424881		√			[68]
*ASIP*	rs6058017		√		√	[71, 72, 74, 118]
	rs4911442		√		√	[40, 118]
*DCT*	rs1325611		√			[49]
	rs1407995		√			[40, 49, 74]
	rs9516418		√			[49]
	rs3782974		√			[50]
	rs2031526		√			[38]
*GRM5*	rs10831469		√			[68]
*HERC2*	rs79097182			√		[60]
*IRF4*	rs12203592		√	√		[40, 118]
	rs1540771			√		[60, 118]
*KITLG*	rs1881227		√			[56, 119]
	rs642742		√			[61, 118]
	rs12821256			√		[40, 60, 118]
*MC1R*	rs885479		√	√		[40, 74, 118, 119]
	rs1805005		√	√		[29, 74, 118, 120]
	rs1805006		√	√		[74, 118]
	rs1805007		√	√		[57, 74, 118, 121]
	rs1805008		√	√		[57, 74, 118]
	rs1805009			√		[118]
	rs2228479		√	√		[74, 107, 118]
	rs2353688			√		[60]
	rs146972365			√		[60]
	rs8063160			√		[60]
	rs11547464		√		√	[118]
	rs1110400		√	√		[118]
*OCA2*	rs12913832		√		√	[23, 40, 68, 118]
	rs1800404		√			[48]
	rs1800407		√		√	[118]
	rs1800414		√		√	[40, 47, 50, 118, 122]
	rs74653330		√			[40, 48]
	rs150335311		√			[48]
	rs1448484		√			[50]
	rs1667394		√			[57]
*SLC24A4*	rs12896399		√	√	√	[57, 118]
	rs8014907			√		[60]
*SLC24A5*	rs1426654		√			[17, 23, 40, 61, 68, 72, 75, 118]
	rs2470102		√		√	[68]
*SLC45A2*	rs16891982		√			[17, 18, 21, 23, 27, 40, 50, 61, 112, 118]
	rs26722		√			[18, 27, 118]
	rs11568737		√			[48]
	rs35395		√			[68]
*TPCN2*	rs72930659			√		[60]
	rs35264875		√			[118]
	rs3829241		√			[118]
*TYR*	rs1042602		√			[40, 57, 68, 118]
	rs1393350		√			[57]
	rs13312741		√			[48]
	rs10831469		√			[68]
	rs1126809		√			[40, 118]
	rs1800422		√			[118]
*TYRP1*	rs2733832		√		√	[49, 118]
	rs2733831		√			[61]
	rs10809814		√			[49]
	rs2762464		√			[50]
	rs1408799		√		√	[40, 118]

The derived allele is associated with pigmentation in European populations

The derived allele is associated with pigmentation in East Asian populations
